# Development and Construct Validation of a Food-Based Diet Quality Score Using Image-Based Food Records

**DOI:** 10.3390/nu18101622

**Published:** 2026-05-20

**Authors:** Amira Hassan, Satvinder S. Dhaliwal, Christina M. Pollard, Andrea Begley, Anthony P. James, Rajshri Roy, Samara Legrand, Tracy A. McCaffrey, Deborah A. Kerr

**Affiliations:** 1 Curtin School of Population Health, Curtin University, Perth, WA 6102, Australia; amira.hassan@curtin.edu.au (A.H.); s.dhaliwal@curtin.edu.au (S.S.D.); c.pollard@curtin.edu.au (C.M.P.); a.begley@curtin.edu.au (A.B.); t.p.james@curtin.edu.au (A.P.J.); 2 Curtin Medical Research Institute, Curtin University, Perth, WA 6102, Australia; 3 Office of the Provost, Singapore University of Social Sciences, 463 Clementi Road, Singapore 599494, Singapore; 4 Health & Social Sciences, Singapore Institute of Technology, 1 Punggol Coast Road, Singapore 828608, Singapore; 5 Obstetrics & Gynaecology Academic Clinical Program, Duke NUS Medical School, National University of Singapore, 8 College Road, Singapore 169857, Singapore; 6 Enable Institute, Faculty of Health Sciences, Curtin University, Perth, WA 6102, Australia; 7 Discipline of Nutrition and Dietetics, Susan Wakil School of Nursing and Midwifery, Faculty of Medicine and Health, The University of Sydney, Sydney, NSW 2006, Australia; rajshri.roy@sydney.edu.au; 8 Charles Perkins Centre, The University of Sydney, Sydney, NSW 2006, Australia; 9 Department of Nutrition, Dietetics and Food, Monash University, Melbourne, VIC 3168, Australia; samara.legrand@monash.edu (S.L.); tracy.mccaffrey@monash.edu (T.A.M.)

**Keywords:** diet quality, diet quality score, image-based dietary assessment, construct validity, mobile food record, adults

## Abstract

**Background/Objectives**: Diet quality indices (DQIs) are commonly used to evaluate the alignment of dietary intake with national dietary guidelines. However, few methods have been developed to apply DQIs to image-based dietary assessment tools. This study aimed to develop a novel food-based Diet Quality Score (DQS) and examine its construct validity in Australian adults aged 18–65 years living with a higher weight (BMI 30–45 kg/m^2^), using the validated Healthy Eating Index for Australians 2013 (HEIFA-2013) for comparison. **Methods**: This cross-sectional study assessed dietary intake in 260 adults (mean age 48 ± 12 years; BMI 35.0 ± 4.2) collected over 4 days using the mobile Food Record (mFR™) and scored using the HEIFA-2013 and DQS (both scored out of 100). Descriptive statistics summarised participant characteristics with DQS and HEIFA-2013 scores. Construct validity was examined by assessing the associations between sociodemographic, health behaviour, anthropometric, and clinical blood marker variables and DQS and HEIFA-2013 scores. Multivariable binary logistic regression identified variables associated with being in the lowest diet quality tertile for both DQIs. **Results**: The mean scores were 47.4 ± 8.7 for the DQS and 52.0 ± 8.6 for HEIFA-2013. Both scores were positively associated with age (DQS: *r* = 0.216, *p* < 0.001; HEIFA-2013: *r* = 0.265, *p* < 0.001) and attention paid to the health aspects of their diet (DQS: *p* < 0.023; HEIFA-2013: *p* < 0.010). Increasing age (OR = 0.958, 95% CI: 0.932–0.985, *p* = 0.003) and moderate versus low physical activity (OR = 0.40, 95% CI: 0.21–0.80, *p* = 0.009) decreased odds of being in the lowest diet quality tertile for both DQI scores. **Conclusions**: The DQS demonstrated acceptable construct validity, providing a valid method for using image-based dietary assessments to evaluate diet quality in Australian populations.

## 1. Introduction

In population-based health research, simplifying the complexity of dietary intake into summary measures is a practical strategy for representing overall diet quality [1]. Diet quality indices (DQI) achieve this by applying an “a priori” scoring framework to dietary intake data. These frameworks are based on predefined dietary recommendations, such as national dietary guidelines, to quantify intake and convert it into a single diet quality score, enabling evaluation of how well the captured intake aligns with these recommendations [1,2]. Higher diet quality scores have been consistently associated with improved health outcomes in longitudinal studies, such as reduced risk of cardiovascular disease, type 2 diabetes, and all-cause mortality [3,4]. This capacity to quantify diet-health relationships enhances interpretability for the general population [5], which makes DQIs valuable tools for monitoring population dietary intakes and informing evidence-based public health interventions [3,5].

The construction of DQI frameworks is primarily achieved through three main methods: (1) food or food-group intakes, (2) nutrient intakes, or (3) a combination of both [6]. Food-based DQIs offer greater practicality, as they can better capture the complexity of dietary patterns [5] and the potential synergistic health effects of consuming whole foods in combination [7]. They are also more aligned with public health messaging, as national dietary guidelines emphasise the consumption of a variety of whole foods as part of a healthy dietary pattern rather than individual nutrients [8], making them useful for nutrition surveillance and program evaluation [9].

As DQIs are calculated from dietary intake data obtained via dietary assessment methods, this determines the type of dietary information that can be captured and subsequently scored. This indicates that DQIs should be tailored to suit the specific dietary assessment method employed [10], because indices designed for one assessment method can lead to substantial differences in diet quality classification when applied to data from another [11]. Dietary assessment methods used to derive DQIs have varied [12]; however, food frequency questionnaires are most commonly employed [1,12] because they are often the most practical for large-scale epidemiological studies [13]. Although food frequency questionnaires are useful for assessing habitual dietary intake patterns by asking about the consumption frequency of specific food items or groups over a specified period, the information they capture is limited by the pre-specified food list [14,15]. Food records can provide more detailed information on food items consumed [16]. However, they can increase the burden not only on participants—if they are required to manually log detailed intake data through paper diaries or digital systems [16,17]—but also on researchers who must perform detailed manual coding of the captured data [16]. Recent technological advances, particularly around image-based approaches using mobile devices, can help address these limitations.

Use of mobile devices for assessing dietary intake has become increasingly common [18,19]. Individuals can use applications on their smartphones, such as the mobile Food Record (mFR™), to capture images of consumed items, which can reduce participant burden while maintaining the accuracy of captured dietary intake data [20,21]. This image-based approach is particularly well-suited for use with food-based DQIs, which only require food-level data rather than detailed nutrient calculations [22]. Consequently, they can be applied directly to dietary intake data derived from food images, such as those captured using the mFR™. As dietary assessment increasingly shifts toward the use of artificial intelligence (AI) applications to evaluate dietary intake from food images [23], the combination of image-based assessment methods and food-based DQIs can facilitate scalable and automated diet quality evaluation. While methodological challenges remain, such as food misclassification, portion size estimation, and reliance on food image quality, this approach has the potential to reduce the burden on participants and researchers associated with dietary data collection and analysis [24,25].

Despite this potential, the integration and validation of image-based assessment with DQIs remain limited, as most DQIs were developed and validated using conventional rather than mobile or image-based dietary assessment methods. While some DQIs may be adaptable, few have been explicitly designed or validated for use with image-based dietary assessment tools. To date, only one DQI—the Healthy and Sustainable Diet Index (HSDI)—has been developed specifically to collect dietary data with the mFR™ [26]. However, it was designed to assess adherence to a healthy and sustainable diet among a sample of young adult Australians. Components of the HSDI were selected and differentially weighted based on the strength of evidence linking dietary behaviours to human health and environmental impact, as outlined in the Australian Dietary Guidelines [27]. No DQI has been designed or validated for use with the mFR™ that provides a focused assessment of healthy eating alignment with the Australian Dietary Guidelines.

Developing a food-based index compatible with image-based dietary records could support both public health monitoring and personalised dietary guidance. A simple score-based summary of dietary intake can enable comparison of diet quality across population groups, while minimising respondent burden through image-based data collection. It can also help identify food group consumption patterns that are easily understood by lay individuals and practitioners, thereby supporting practical, food-based recommendations to improve diet quality.

To support the adoption of such an index in research and practice, it is essential to establish its validity, demonstrating that it appropriately and meaningfully captures diet quality. Construct validity is the most used approach for validating DQIs in Australian contexts [1,28]. It examines whether DQIs exhibit expected associations with sociodemographic variables and health markers [1,28]. Comparing the construct validity of a newly developed index with that of an established, validated index provides context for determining whether the new index demonstrates comparable and theoretically expected association patterns with relevant variables. Among the twenty-four identified DQIs used to assess diet quality in Australian adults [1], the Healthy Eating Index for Australians-2013 (HEIFA-2013) was selected as the most appropriate comparator to evaluate the construct validity of a newly developed index designed to assess adherence to the 2013 Australian Dietary Guidelines. This is because HEIFA-2013 was the only DQI to evaluate intake alignment with the 2013 Australian Dietary Guidelines and use a scoring framework methodologically compatible with image-based dietary assessment outputs. HEIFA-2013 has also previously demonstrated construct validity in young Australian adults, showing expected associations of diet quality with sociodemographic factors [29].

The aims of this study are therefore: (1) to develop a food-based DQI, the Diet Quality Score (DQS), that evaluates intake alignment with the Australian Dietary Guidelines (2013) using dietary intake data collected using the mFR™ by Australian adults aged 18–65 years living with a higher weight (BMI 30–45 kg/m^2^), and (2) to assess the construct validity of this DQI by examining its associations with sociodemographic characteristics and health markers, using HEIFA-2013 as a comparative reference.

## 2. Materials and Methods

### 2.1. Study Sample

This cross-sectional research was conducted using baseline data from participants in the Connecting Health and Technology 2 (chat2) trial. The chat2 trial was a 1-year randomised controlled trial comparing a digital dietary intervention with standard care in Australian adults aged 18–65 years living with a higher weight (BMI 30–45 kg/m^2^). Participants were eligible if they owned a mobile telephone with internet access and could attend all visits at the study centre in metropolitan Perth. Exclusion criteria included medical conditions or treatments likely to influence dietary intake or weight management (e.g., insulin-dependent diabetes, severe cardiovascular disease, palliative illness, eating disorders, or medical dietary restrictions); current dietetic counselling; current or planned use of weight-loss medications or surgery within 12 months; pregnancy, breastfeeding, or planned pregnancy or breastfeeding; and inability to participate in telehealth dietary consultations. Participants were recruited through letters sent to individuals randomly selected from the Federal Electoral Roll, a compulsory system for Australians aged 18 years or older. They were selected from suburbs within the Perth metropolitan area to provide representation across socio-economic status. Other recruitment methods supplemented the mailout and included unaddressed mailbox delivery to residential street points, flyers in hospitals, and social media advertising across Perth, Western Australia, between July 2023 and August 2024, to support broader reach across community and clinical settings.

The sample size was determined a priori based on a power calculation indicating that 342 participants would be required to detect a between-group difference of at least 5% body weight at 12 months, at 90% power and 5% significance level [30]. The intended recruitment target was not reached. A total of 280 participants were randomised, of whom 260 were included in the final analysis. This is acknowledged as a limitation that may have reduced statistical power to detect smaller between-group differences. Further details about the study protocol have been previously published [30].

The trial was registered on the Australian and New Zealand Clinical Trials Registry (ACTRN12622000803796) and approved by the Curtin University Human Research Ethics Committee (HRE2022-0059) and the Department of Health WA Human Research Ethics Committee (RGS0000005490).

### 2.2. Sociodemographic Characteristics and Health Markers

Participants completed a baseline survey that assessed sociodemographic characteristics, including age, gender, household income, smoking status (including vaping), and ethnicity. Socioeconomic status was assessed using the Index of Relative Socio-Economic Advantage and Disadvantage (IRSAD) based on participants’ residential postcodes [31]. Food security was assessed using the US Department of Agriculture’s Food Sufficiency Question [32].

Weight loss history was assessed using items from the Weight-Loss History tool, which inquired about previous weight loss attempts with and without assistance [33]. Attention paid to the health aspects of diet and cooking skills were assessed using “Feelings about health aspects of food and diet” and “Self-assessment of cooking skills” items from the Western Australian Department of Health’s Nutrition Monitoring Survey Series [34], respectively. Physical activity was assessed using the short form of the International Physical Activity Questionnaire (IPAQ) and quantified in terms of Metabolic Equivalent of Task (MET) minutes per week. Calculated MET minutes were then classified into different categories of physical activity levels: low/inactive, moderate, and high/active [35]. Across all variables, only available data were analysed; missing values were not imputed.

During the initial study visit, participants’ anthropometric measurements were taken by trained researchers. Body weight was measured to the nearest 0.1 kg using daily calibrated digital scales, and a stadiometer was used to measure height (in cm) to the nearest 0.5 cm. Body composition was assessed using a whole-body dual-X-ray-absorptiometry (DXA) scan of regional or total body fat (including visceral body fat). The DXA machine (Hologic Inc., Marlborough, MA, USA) was calibrated per manufacturer protocol before each session. Fasting blood samples were analysed according to standard laboratory procedures for lipid and glucose profiles, including total cholesterol (<5.0 mmol/L), triglycerides (<2.0 mmol/L), high-density lipoprotein cholesterol (HDL-C; >1.0 mmol/L), low-density lipoprotein cholesterol (LDL-C; <3.0 mmol/L), non-high-density lipoprotein cholesterol (non-HDL-C; <3.9 mmol/L), total cholesterol/HDL ratio (<3.5), fasting glucose (3.5–5.4 mmol/L), and glycated haemoglobin (HbA1c; <6.5%) [36,37,38]. Reference ranges are provided in parentheses for clinical context.

### 2.3. Dietary Assessment

Participants recorded their dietary intake using the mFR™ application (TADA3.3.1.ipa and 1.0.chat2i) for smartphones to capture dietary intake through images of food and beverage items consumed [39,40,41,42,43]. The application was installed on participants’ mobile devices by a research dietitian during an initial study orientation visit. Participants received training on how to use the application, along with a printed instruction sheet that could be taken home for later reference. Participants were asked to take before-and-after images of all foods and beverages consumed, excluding dietary supplements, for four consecutive days, including one weekend day, before their subsequent study visit. In cases where an after-image was missed, the mFR™ application indicated that the eating occasion was incomplete and prompted users to capture an image to complete the record and initiate a new eating occasion. Each image needed to include a fiducial marker, a credit card-sized reference checkerboard, to more accurately estimate the portion size of items consumed that were captured in the images [40]. After taking each image pair, participants were instructed to label the image content on their phones by selecting a food or beverage ‘mini label’ from a drop-down list within the mFR™ application. The mini labels were condensed versions of the full identification codes for food items from the Australian Food and Nutrient Database (AUSNUT 2011-13) [44]. For example, participants were able to select the ‘chicken and salad sandwich’ mini label instead of the full AUSNUT identification code ‘sandwich or roll, filled with chicken & salad’. Participants could label meal images immediately after each pair was taken or wait until all meal occasions had been captured. All images were automatically uploaded to a dedicated secure cloud server via Wi-Fi or 4G/5G network for storage.

At a follow-up study visit one week later, a research dietitian reviewed the 4-day mFR™ images with participants using a Web Platform that accessed the cloud server where the images were stored. Prior to data collection, research dietitians involved in the dietary review process received standardised training on dietary interview procedures, including how to probe for food preparation methods, portion sizes, and missing items, to minimise inter-rater variability. During the review, as standard procedure, the dietitian probed for missing data and clarified image content with participants. Participants who recorded fewer than two days or provided no images of dietary intake were excluded from dietary analysis (*n* = 8). For participants with valid dietary data, the consumed food and beverage items were identified from the mFR™ images, with the information confirmed and verified by the labels participants applied to their images and by notes recorded by the dietitian during the review process. A single trained dietitian analyst entered the data into Foodworks [45], a nutrition analysis software linked to the AUSNUT 2011-13 database [44], following predefined data entry procedures. Inter- and intra-observer variability were therefore not formally assessed. Portion sizes were estimated using the fiducial marker in each image as a reference and supplemented by information from the dietitian’s review when needed. Evidence from a controlled feeding study indicates that analyst-estimated intakes align well with true average intakes and are comparable to participant-reported methods [46]. Once all food items and their corresponding portion sizes were entered into Foodworks, data were mapped to AUSTNUT 2011-13 [38] to derive food intake and nutrient composition data. These data were then exported into Microsoft Excel for further analysis [47].

### 2.4. Dietary Data Processing for DQS

The exported Foodworks file was matched to two additional Excel databases. Both databases contained all items found in the AUSNUT 2011-13 database [38]. The first database was the Australian Health Survey—Discretionary Food List [48]. This database classifies items in the AUSNUT database as discretionary if they are energy-dense, high in added sugars, saturated fat, added sodium, or alcohol, and low in fibre [49]. This classification allowed for all discretionary items consumed by participants to be identified, including discretionary foods, sugar-sweetened beverages (SSBs), and alcoholic beverages.

The second database was the Australian Health Survey—Australian Dietary Guidelines [50]. This database specified the mean amount of each Australian Dietary Guideline food group contained in all AUSNUT 2011-13 food and beverage items per 100 g. This information facilitated the identification of food groups present in each item consumed by participants, with food group servings being determined by the portion of the food or beverage item consumed (e.g., a chicken and salad sandwich would be disaggregated into its components, with servings allocated to the meat and alternatives (chicken), vegetable (salad), and grain (cereal) food groups according to the portion of each consumed).

The total portion consumed from each food group across all recorded days was summed for each participant. Food and beverage items identified as discretionary foods, SSBs, and alcoholic beverages were not included in food group serving calculations. To determine the total number of servings consumed for these items, the Australian Dietary Guidelines serving size recommendations [49] were applied by separately summing the energy content (kJ) of all discretionary foods, SSBs, and alcoholic beverages per participant and dividing each total by 600 kJ. Fruit variety and vegetable variety, defined as the number of unique fruit or vegetable items consumed, were manually determined as separate measures during the process of inputting consumed food and beverage items from captured images into the Foodworks software (Foodworks 10, Xyris Pty Ltd., Brisbane, Australia). The amount of plain water consumed per participant was converted from cups to litres and summed for each participant. To calculate the mean daily intake for each component, all determined servings for the five core food groups (grain (cereal) foods, fruits, vegetables, milk and milk alternatives, and meat and alternatives), discretionary foods, SSBs, alcoholic beverages, fruit variety, vegetable variety, and plain water were divided by the number of days recorded by the participant.

**Figure 1:**
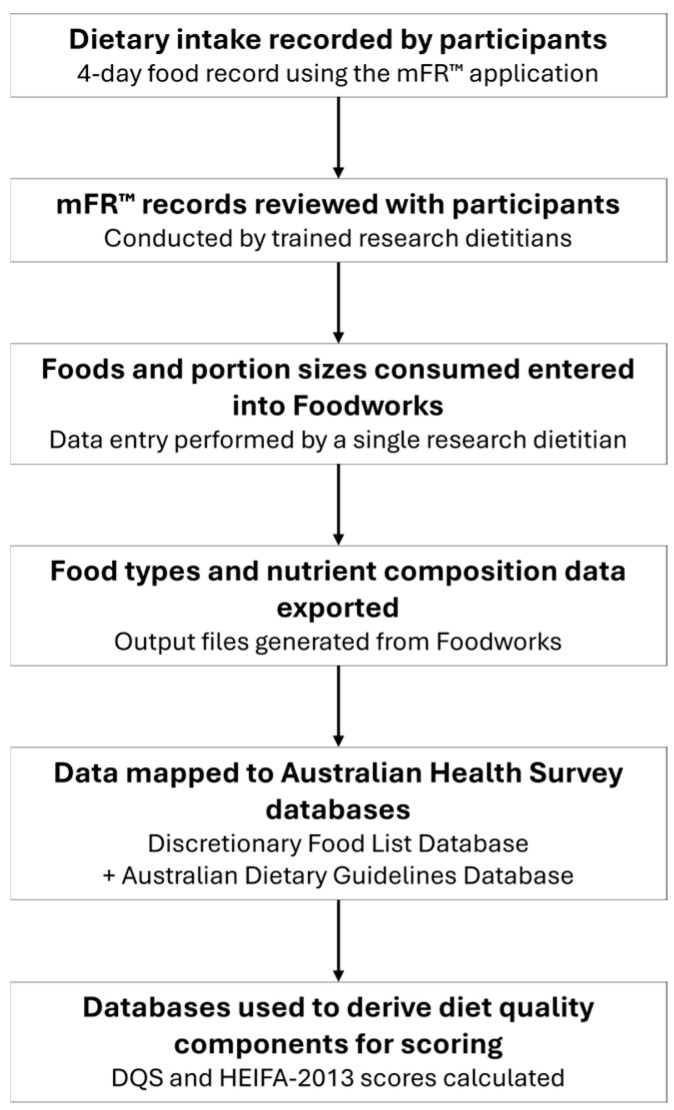
Overview of dietary data collection, processing, and diet quality score derivation.

Data quality checks were conducted. Manual verification of potential outliers was performed across all macronutrients, micronutrients, and vitamins to identify data input errors. All outlier values were cross-referenced with their corresponding mFR™ images to confirm they reflected participants’ actual reported intakes. No manual input errors were detected. The calculated mean daily values of consumed dietary components were then used to calculate HEIFA-2013 and DQSs for each participant. Figure 1 summarises the collection and processing of dietary data to calculate diet quality scores.

### 2.5. DQS Scoring Method

A novel DQI, the DQS, was developed based on the food group recommendations in the Australian Dietary Guidelines-2013 [49], with scoring approaches adapted from two existing DQIs, the HEIFA-2013 and HSDI [10,26]. Both DQIs were developed for use with Australian adults. HEIFA-2013 is a food- and nutrient-based DQI validated using weighed food records and FFQs to assess guideline adherence [10]. HSDI is a food-based DQI applied to mFR™ data to assess environmentally sustainable dietary behaviours consistent with the guidelines [26]. Building on these two approaches, the DQS is the first food-based DQI designed for use with the mFR™ to assess intake alignment with the same dietary guidelines. The scoring criteria for the DQS consisted of 11 components outlined in the Australian Dietary Guidelines-2013. The 11 scoring components include the five core food groups—grain (cereal) foods, fruits, vegetables, milk and milk alternatives, and meat and alternatives—as well as plain water, fruit variety, vegetable variety, alcohol, sugar-sweetened beverages (SSBs), and discretionary foods (see Table 1).

The DQI scores ranged from 0 to 100 to simplify the interpretation of total diet quality scores. Nine components (the five core food groups, discretionary foods, SSBs, alcohol, and plain water) were each scored from 0 to 10 points to reflect the Australian Dietary Guidelines’ primary emphasis on meeting recommended servings of core food groups and limiting discretionary choices. Two components (fruit variety and vegetable variety) were each scored from 0 to 5 points, as even though dietary variety is important for nutritional adequacy, it is intended to complement achieving adequate total servings of core food groups.

Points were allocated to each component based on how closely participants’ mean daily servings aligned with Australian Dietary Guideline recommendations (Table 1). A proportional scoring approach was used to assign scores between the minimum and maximum thresholds for each component. For components with specified serving recommendations (e.g., vegetables, fruit, grains), scores were calculated as the proportion of recommended servings consumed. For variety components (fruit variety and vegetable variety), scores were based on the mean number of unique types consumed per day.

Three scoring frameworks were applied, each based on the relationship between dietary components and health outcomes. Adequacy components (vegetables, vegetable variety, fruit, fruit variety, milk and alternatives, and plain water) were scored such that higher intakes corresponded to higher scores, with maximum points awarded when participants met or exceeded the recommended daily serving amounts. These food groups were selected as adequacy components because of two considerations: evidence of health benefits associated with adequate intake [51,52] as well as the goal of encouraging consumption in the context of typical underconsumption in the Australian adult population [53]. Plain water was included to encourage consumption as a replacement for discretionary beverages, as less than fifty percent (48.3%) of daily beverage intake in Australian adults comes from plain water [53].

Optimal range components (grain (cereal) foods, and meat and alternatives) received maximum points only when intake fell within the recommended range, with scores of zero assigned for inadequate consumption and for intakes that exceeded the recommended number of serves. This framework was applied to selected components because of the dual concerns of inadequate intake and potential harm from excessive consumption, particularly considering known adverse health effects associated with high intakes of refined grains and red meat [54,55,56]. Given that refined grains and red meat are the predominant contributors within these food groups in the Australian adult population (63.7% and 37.9% of total intakes, respectively) [53], this approach was designed to encourage wholegrain consumption and dietary variety and discourage overconsumption.

Moderation components (discretionary foods, SSBs, and alcoholic beverages) were assigned reverse scoring, whereby lower intakes received higher scores and zero consumption received the maximum score. Reverse scoring reflects dose–response evidence indicating increased disease and mortality risk at higher consumption levels [57]. Reverse scoring was also incorporated as an intervention feature in the chat2 study to encourage reduced consumption of these items, supporting the primary goal of body weight reduction [30].

### 2.6. Dietary Data Processing for HEIFA-2013

The Foodworks output file, mapped to the AUSNUT 2011-13 database [38], containing both food intake and nutrient composition data, was processed through the HEIFA-2013 automated scoring system by Author 7 [58,59]. This system disaggregates each consumed item into its individual ingredient components, with each ingredient assigned to a corresponding food group (e.g., a chicken and salad sandwich would be disaggregated into chicken, salad, and bread, and then allocated to the meat and alternatives, vegetable, and grain (cereal) food groups, respectively). The portion sizes of each ingredient are then used to calculate the serving size contribution for that specific food group. Nutrient intakes are calculated directly from the nutrient composition data available in the Foodworks file, based on AUSNUT 2011-13 data, for the food items consumed. Data quality checks were conducted, with no errors identified.

### 2.7. HEIFA-2013 Scoring Method

A detailed description of the HEIFA-2013 scoring methodology has been published elsewhere [10]. In brief, the HEIFA-2013 was developed by Australian researchers to assess dietary intake alignment with the 2013 Australian Dietary Guidelines [49]. There are 15 scored components in HEIFA-2013, comprising food groups and nutrients; the scoring encompasses adequacy and moderation approaches. The 10 adequacy components include fruits (quantity and variety), vegetables (quantity and variety), water, cereal/bread, whole grains, milk and milk alternatives, meat and protein alternatives, and unsaturated fats. The five moderation components with reverse scoring applied include discretionary foods, alcohol, sodium, added sugars, and saturated fat. Each component is scored from 0 to a maximum of either 5 or 10 points, resulting in a total score ranging from 0 to 100, with higher scores indicating better diet quality. The basis of the scoring for most components is the amount consumed per day (e.g., serves/day), whereas four components use proportion-based scoring, including wholegrains (daily proportion of wholemeal/wholegrain bread relative to total bread), added sugars (percentage of daily energy intake), saturated fat (percentage of daily energy intake), and water (percentage of daily beverage intake). These scoring criteria were applied to the processed dietary intake data from the Foodworks file to calculate individual HEIFA-2013 scores for each participant. Table 2 provides a comparative summary of the scoring frameworks for HEIFA-2013 and DQS.

### 2.8. Statistical Analyses

All statistical analyses were conducted using SPSS Version 30 [60], with statistical significance set at *p* < 0.05. To account for potential misreporting of dietary intake, the Schofield equation was used to calculate each participant’s basal metabolic rate (BMR) based on their individual weight, gender, and age [61]. The ratio of energy intake to BMR (EI:BMR) was then calculated. Participants in the top and bottom 2.5% of the EI:BMR distribution were excluded from further analysis (*n* = 12), consistent with the statistical principles underlying the Goldberg cut-off method, which considers values outside the 95% confidence limits to be physiologically implausible [62]. This method has been applied in previous studies, including the HEIFA-2013 validation study [10].

Descriptive statistics were calculated for all participants, including sociodemographic characteristics and health markers. Mean total diet quality scores across the whole sample were reported for the DQS and HEIFA-2013 (mean ± SD), along with mean scores for each dietary component within both scoring systems (mean ± SD). Mean difference and correlation between the DQS and HEIFA-2013 total scores were assessed using paired *t*-tests and Spearman’s correlation, respectively.

The construct validity of the DQS was assessed by examining its relationships with sociodemographic variables and health markers. Spearman’s correlations and scatterplots were used to examine the associations between continuous variables and diet quality scores. For categorical variables, mean diet quality scores were compared across groups using independent *t*-tests (for two categories) or one-way ANOVA (for more than two categories), with normality and homogeneity of variance assumptions assessed, and results presented as *p*-values and boxplots. These same analyses were conducted for HEIFA-2013 scores to provide a comparative context.

Regression analyses were conducted using multivariable logistic regression, with relevant sociodemographic and health variables considered simultaneously to account for potential confounding. Multicollinearity among predictors was assessed. In instances where multicollinearity was present, only one representative variable was retained in the final model. Multivariable binary logistic regression identified sociodemographic and health predictors of poor diet quality among participants, as measured by both diet quality indices. Participants were categorised into tertiles (lowest, middle, and highest) based on their total diet quality scores for each scoring method using the SPSS rank function. The use of quantiles for continuous variables is widely employed in nutrition studies [63], as it simplifies interpretation by grouping participant diet quality scores into meaningful categories. The approach ensures a clear and straightforward presentation of results while avoiding the assumption of a linear relationship between diet quality scores and study outcomes [64]. After participants were categorised into tertiles, a binary variable was created to indicate whether participants were classified in the lowest tertile by both DQIs. The combined lowest tertile variable was used as the outcome in the logistic regression model. Results are presented as odds ratios with 95% confidence intervals and *p*-values.

## 3. Results

**Figure 2:**
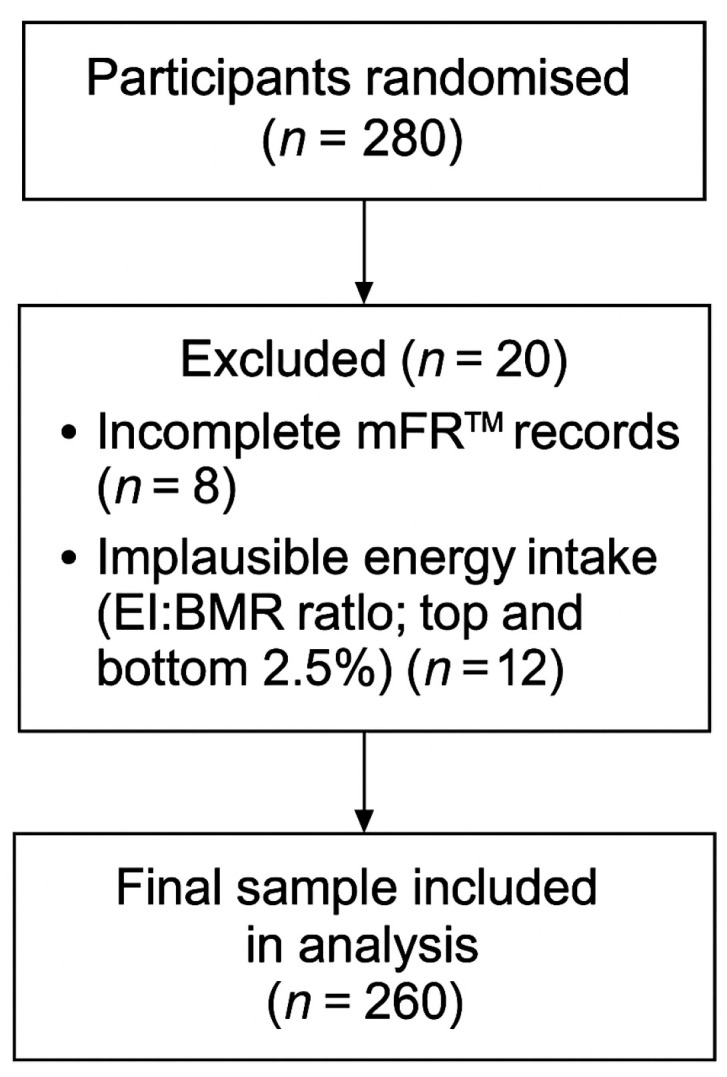
Participant flow diagram.

The dietary intake data of 280 participants were initially collected. Eight participants were excluded because they had fewer than two days of recorded intake or no images of dietary intake. An additional 12 participants were excluded because they fell within the top and bottom 2.5% of EI:BMR ratios. Thus, the final sample size for diet quality validation and data analysis was 260 participants (Figure 2). The average number of completed dietary record days was 3.7/4.

Table 3 presents the sociodemographic and health characteristics for the 260 participants. The mean age was 48 ± 12 years, with a mean BMI of 35.0 ± 4.2 kg/m^2^. Most had enough of the kinds of food they wanted (82.5%), self-identified as White (75.6%), ‘took a bit of notice of the health aspects of food’ (72.6%), held a university bachelor’s degree or higher (64.7%), and never smoked (56.0%). Fasting blood lipid and glucose values clustered near reference ranges, with only minor elevations observed.

Table 4 presents the mean diet quality scores for both DQIs, including total scores and dietary component scores. The overall diet quality score out of 100 for the DQS was 47.4 ± 8.7, and for HEIFA-2103, it was 52.0 ± 8.6. There was a significant mean difference of −4.7 points between the DQS and HEIFA-2013 scores (95% CI: [5.8, −3.5], *p* < 0.001), and a moderate positive correlation of *r* = 0.357 (*p* < 0.001), suggesting reasonable agreement between the two indices while reflecting differences in their component structures, scoring approaches, and component weightings. Table 4 also presents mean scores for each dietary component under the DQS and HEIFA-2013; however, statistical comparisons were limited to total scores due to these structural and conceptual differences between the indices. Consequently, comparisons of mean differences and correlations between individual components were not conducted.

Table 5 presents descriptive subgroup comparisons of both DQIs across categorical sociodemographic variables, highlighting similar trends across both indices. HEIFA-2013 consistently showed higher mean values across all subgroups compared with the DQS, reinforcing the observed mean difference between the two scoring methods.

**Figure 3:**
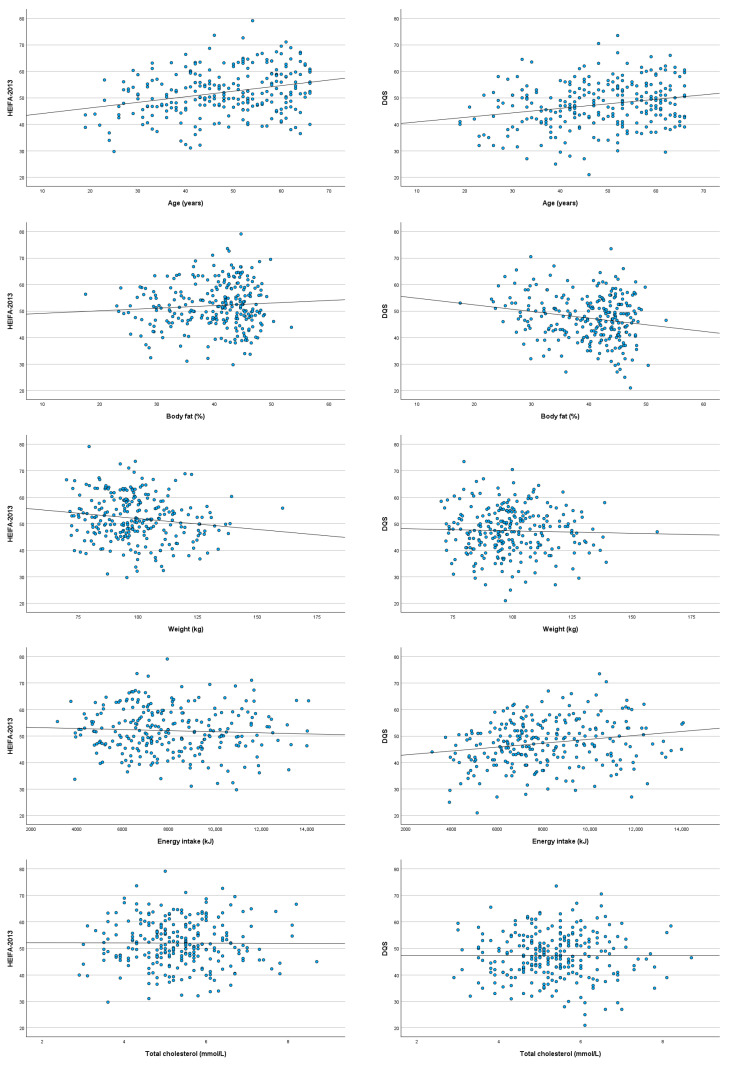
Scatterplots of HEIFA-2013 and DQS with relevant continuous sociodemographic variables and health markers: age, body fat composition, weight, energy intake, and total cholesterol.

Figure 3 presents scatterplots showing the bivariate relationships between diet quality scores and relevant continuous variables. Both scores showed positive associations with age (DQS: *r* = 0.216, *p* < 0.001; HEIFA-2013: *r* = 0.265, *p* < 0.001). Body fat percentage showed a weak inverse relationship with the DQS (*r* = −0.144, *p* = 0.02) and a negligible association with HEIFA-2013 (*r* = 0.037, *p* = 0.549). Weight showed a negligible association with the DQS (*r* = 0.009, *p* = 0.884) and a weak inverse relationship with HEIFA-2013 (*r* = −0.144, *p* = 0.021). Average energy intake was weakly positively associated with the DQS (*r* = 0.194, *p* = 0.002) and had a negligible association with HEIFA-2013 (*r* = −0.060, *p* = 0.337). Total cholesterol levels displayed negligible associations with both scores (DQS: *r* = 0.018, *p* = 0.774; HEIFA-2013: *r* = −0.021, *p* = 0.737). All other clinical metabolic markers and anthropometric measures showed weak to negligible and non-significant associations with both scores.

**Figure 4:**
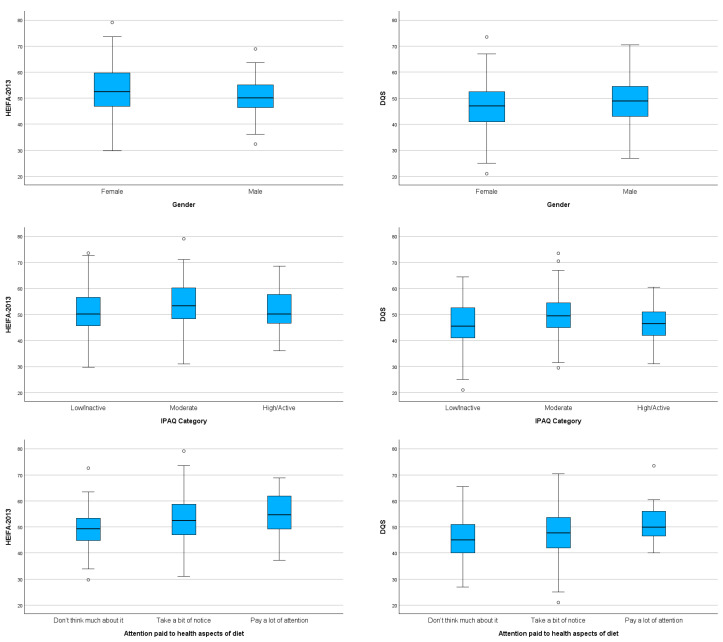
Boxplots of HEIFA-2013 and DQS with relevant categorical sociodemographic variables: gender, IPAQ category, and attention paid to the health aspects of diet.

Figure 4 presents boxplots examining the relationships between diet quality scores and relevant categorical variables. Attention paid to the health aspects of diet showed consistent associations across both scores, with greater consciousness associated with higher diet quality scores (DQS: *p* < 0.023; HEIFA-2013: *p* < 0.010). MET physical activity categories showed similar patterns across both scores, with moderately active participants obtaining higher diet quality scores than those who were less or more active (DQS: *p* = 0.008; HEIFA-2013: *p* = 0.066). Gender displayed significant but contrasting patterns between scores, with females scoring lower than males on the DQS (*p* = 0.047) but higher on the HEIFA-2013 score (*p* = 0.024). For the remaining sociodemographic variables, both scores showed similar, largely non-significant patterns. Although occasional differences in statistical significance appeared between scores for individual variables (e.g., MET physical activity categories reached significance for the DQS but not for HEIFA-2013), visual inspection of boxplots and descriptive statistics in Table 5 confirmed consistent trends.

Multivariable binary logistic regression was conducted to identify variables associated with being classified in the lowest diet quality tertile, as determined by both DQIs (Table 6). All variables presented in Table 4 and Table 5 were initially considered for inclusion in the multivariable models. Weight and lipid markers were excluded from the final models due to collinearity, with total cholesterol retained as the sole lipid marker. The global fit index for the final model was Nagelkerke R^2^ = 0.207, with an overall correct classification rate of 64.2%.

Older age was associated with 4% lower odds of being in the lowest diet quality tertile (OR = 0.957, 95% CI: 0.931–0.984, *p* = 0.002), while higher total cholesterol levels were associated with 39% higher odds (OR = 1.385, 95% CI: 1.031–1.861, *p* = 0.031). Moderate physical activity was associated with 57% lower odds of being in the lowest diet quality tertile compared with low physical activity (OR = 0.435, 95% CI: 0.225–0.843, *p* = 0.014). Attention paid to the health aspects of diet showed a significant association, with participants who reported not giving much thought to their diet having nearly 20 times the odds of being in the lowest diet quality tertile compared with those who paid a lot of attention (OR = 18.117, 95% CI: 2.095–156.653, *p* = 0.008).

## 4. Discussion

As image-based dietary assessment becomes more prevalent in research and practice, DQIs designed and validated for use with these methods are needed to advance diet quality research. Food-based DQIs are particularly well-suited for use with image-captured dietary intake data and will become increasingly important as automated approaches to dietary assessment based on food images continue to develop. Although most DQIs have been developed and validated using non-digital dietary assessment methods, several widely used diet quality indices, including the Global Diet Quality Score and the Mediterranean Diet Score, have been operationalised for use with digital-based methods [65,66]. These adaptations demonstrate the feasibility of integrating diet quality evaluation with emerging technologies. Despite these advancements, few have been explicitly designed or validated for use with image-based dietary assessment tools. No validated food-based DQI that solely assesses healthy eating patterns against the Australian Dietary Guidelines-2013 and can be applied directly to food images without requiring nutrient calculations has been developed. This study has addressed the gap by developing the DQS, a novel food-based DQI that assesses alignment of healthy intake with the Australian Dietary Guidelines-2013 and can be applied directly to image-based dietary records. Construct validity was examined in 260 Australian adults living with higher weight and demonstrated through expected associations with sociodemographic and health indicators. Scores from the DQS showed similar directional relationships to those of the HEIFA-2013, a validated food- and nutrient-based index, across multiple variables, and identified shared variables that increased the odds of having diet quality scores in the lowest diet quality tertiles. These findings support the construct validity of the new DQI for diet quality assessment in this study sample.

Both DQIs classified this study sample as having moderate diet quality, with mean scores of 47.4/100 for DQS and 52.0/100 for HEIFA-2013. Although this difference was statistically significant, dietary component scores between the DQS and HEIFA-2013 are not directly comparable because of differences in scoring approaches; however, the similarity in overall diet quality suggests that both tools measure similar underlying dietary constructs in this sample. Consistent with this, both indices indicate low consumption across core food groups. This finding aligns with broader Australian dietary intake patterns documented in the National Nutrition and Physical Activity Survey (NNPAS) 2011-12, which indicate persistently low intakes of the core food groups [53]. Although the NNPAS 2023 food group intake results had been released at the time of writing, the analyses assessing whether consumed intakes meet recommended serving recommendations were still pending. This concordance with population-level surveillance data strengthens confidence in the ability of both tools to characterise and identify dietary intake patterns in Australian adult populations.

Diet quality scores from both DQIs increased significantly with age and were higher among individuals who paid greater attention to the health aspects of their diet, highlighting the role of life stage and personal attitudes in shaping dietary patterns. This aligns with previous research demonstrating that diet quality is poorer among younger adult populations [67,68,69,70], including findings from an earlier study of young Australian adults using HEIFA-2013 [29]. This age-related improvement is suggested to reflect the greater attention older individuals pay to the health aspects of their diet [70]. Similarly, higher scores among those who paid more attention to the health aspects of their diet extend previous diet quality research in Australian populations using the mFR™. This research demonstrated that Australian adults who pay greater attention to dietary health are less likely to consume discretionary items [71] and are less likely to have low diet quality scores [26]. This mirrors broader evidence that those who actively consider health consequences during food selection [72] and pay more attention to food labels [73] achieve better diet quality. The concordance between these two DQIs strengthens confidence in these relationships, suggesting that age and attention paid to the health aspects of diet are important determinants of diet quality.

There were significant but divergent associations between the DQIs and gender. Specifically, males had higher DQSs relative to females, whereas females had higher HEIFA-2013 scores relative to males. These differences likely reflect variations in scoring approaches between the two methods. The DQS, as a food-based index using intake-based scoring, may favour men due to their typically higher food intakes compared with women [74,75]. HEIFA-2013 may favour women, as its scoring components include both the types of foods consumed (e.g., discretionary items) and aspects of their nutrient composition (e.g., added sugars). Research has shown that women often select and consume food items with higher nutrient density, while avoiding more processed and energy-dense options [76,77,78,79], which are often high in added sugars, sodium, and saturated fat. Consequently, HEIFA-2013’s combined focus on food types and nutrient composition may allow the score to reflect women’s more health-conscious choices through multiple pathways. Although speculative, these divergent gender associations suggest that the DQIs may capture complementary rather than interchangeable aspects of diet quality. This should be explored in future studies and considered when selecting and interpreting scores from these tools.

Four variables were associated with being in the lowest diet quality tertile across both DQIs. Older age was associated with reduced odds, as was moderate physical activity when compared with low physical activity. Conversely, higher total cholesterol levels were associated with increased odds, as was low attention paid to the health aspects of diet when compared with greater attention. Findings regarding age and attention paid to the health aspects of their diet reinforce patterns observed in bivariate analyses, strengthening these variables as key drivers of diet quality in this population. Interestingly, low attention paid to the health aspects of diet had the strongest association with being in the lowest diet quality tertile, consistent with a prior Australian-based diet quality study using the mFR™ [26]. The consistent strength of this relationship suggests that assessing attention to dietary health aspects may help identify individuals most likely to benefit from dietary change, supporting the development of more effective, targeted interventions.

Notably, total cholesterol did not show significant bivariate associations with either index; however, it was significantly associated with being in the lowest tertile for both DQS and HEIFA-2013. Previous studies have similarly found differences between diet quality tertiles, with higher total cholesterol levels observed in the lowest diet quality tertiles for other DQIs [80,81,82]. These associations were mostly attributed to greater intakes of energy-dense items, particularly those high in saturated fat, such as discretionary foods [67,70], as well as lower intakes of nutrient-dense ones, including fruits and vegetables [81,83,84]. Our findings are consistent with this pattern, suggesting that total cholesterol may be associated with poorer diet quality in similar populations to this sample. Undertaking component-level analyses examining individual dietary components in relation to lipid markers and assessing and adjusting for lipid-lowering medication use would strengthen the interpretation of this association.

Additionally, moderate physical activity demonstrated similar patterns across both indices but was only significantly associated with the DQS in bivariate analyses. However, it remained significantly associated with not being in the lowest diet quality tertile for both indices in the multivariable model. Engagement in physical activity is generally positively associated with diet quality [70,85,86], as individuals are often motivated by health reasons and may therefore be more likely to avoid energy-dense foods perceived as unhealthy [87]. However, in our sample, high physical activity was less protective against poor diet quality than moderate physical activity. This may reflect differences in dietary behaviours among highly active individuals, whose higher energy requirements increase overall food intake [88], but this additional intake may not be proportionally distributed across core food groups [89,90]. This finding highlights the importance of considering the influence of physical activity engagement on dietary choices and, thus, overall diet quality, warranting further investigation.

This study presents the first food-based DQI specifically designed for use with image-based dietary assessments, evaluating dietary intake alignment with the Australian Dietary Guidelines-2013. This novel approach addresses a gap in dietary assessment methodology, establishing a new reference standard for evaluating diet quality in Australian populations using food images. The food-based focus of the DQS eliminates the need for nutrient-level data, simplifying the scoring process and aligning well with evolving image-based dietary assessment approaches. The DQS framework can also be adapted to different contexts, as is common practice for DQIs [1], to ensure suitability and appropriateness for various research questions and aims. Component weightings can be modified and additional components included as new evidence emerges, particularly with the anticipated release of the updated Australian Dietary Guidelines [91] at the time of writing. This flexibility supports applications with larger and more diverse population groups, age ranges, and settings.

The DQS demonstrated good construct validity, showing similar associations and patterns with anthropometric, health markers, and sociodemographic variables to those observed with HEIFA-2013. In addition, multiple variables were significantly associated with the odds of being in the lowest diet quality tertile for both DQS and HEIFA-2013. These results suggest that despite their methodological differences, both DQIs capture similar constructs of diet quality and can reliably identify individuals who would benefit from dietary intervention.

Additional key strengths of this study include the use of the mFR™ for dietary intake assessment, because it has demonstrated the highest accuracy among four technology-assisted dietary assessment methods for estimating energy intake [46]. This level of accuracy reinforces its suitability for evaluating diet quality. The use of a standardised, image-based dietary assessment protocol also ensured consistent data collection across participants, enhancing the robustness of diet quality evaluation in this study. Furthermore, the inclusion of objective clinical biomarkers supports the construct validity of the DQS beyond self-reported measures. The large sample size also strengthens confidence in the observed associations.

Several limitations should, however, be noted. The cross-sectional design limits exploration of causal or predictive relationships between diet quality and other variables. It should also be noted that the generalizability of observed findings may be limited by the characteristics of our sample, which comprised adults aged 18–65 years living with a higher weight (BMI 30–45 kg/m^2^) recruited in metropolitan Perth, Western Australia. Although we attempted to recruit a population-based sample via the Federal Electoral Roll, respondents may not have been representative of the broader population, particularly as supplementary recruitment methods, such as social media, were also used. Furthermore, participation required smartphone/internet access and in-person attendance at study visits, which may have introduced selection bias favouring individuals with greater digital access and capacity to engage in health research. Consistent with this, the sample included a higher proportion of participants who were women (71%), had higher education (64.7%) and food sufficiency (82.5%), and identified as White (75.6%). Nevertheless, the DQS is grounded in the Australian Dietary Guidelines and uses food-based components, supporting its potential applicability beyond the present sample. It should, however, be noted that the DQS was developed using a single set of age-based Australian Dietary Guideline food-group serve targets, and its performance was not evaluated across different age groups. External validation is therefore needed to confirm its performance in more diverse cohorts, age groups, and other settings.

Furthermore, as with other dietary assessment methods, image-based dietary recording may be subject to misreporting or reactivity bias [92]. Remembering to take an image, particularly snacks, may have also contributed to the misreporting of intake [93,94]. Additionally, we only compared our index with the HEIFA-2013. Although comparisons with additional diet quality indices designed to assess intake in the Australian population may have provided additional insights, HEIFA-2013 was the only index that operationalised the latest version of the Australian Dietary Guidelines for which compatible data were available. It is also worth noting that HEIFA-2013 was originally validated in a younger adult population (18–34 years, with a mean age of 23.5 ± 4.1 years), whereas our sample consisted of adults aged 18–65 years, with a mean age of 48 ± 12 years.

The current dietary analysis process from the mFR™ dietary intake images also imposes a burden on researchers due to the required manual portion size estimation by a trained analyst [18,23]. While this reflects standard practice in image-based dietary assessment, residual error in portion estimation can occur, particularly when foods are partially obscured or the fiducial marker is not visible or positioned correctly. For the DQS, given that component scoring is threshold-based, estimation errors would mainly affect classifications near scoring cut-off points rather than undermine the overall validity of the score. Future advances in automated methods may reduce reliance on trained analysts for portion size estimation. The combination of image-based dietary assessment with a food-based scoring approach is particularly well-suited for automated, AI-driven analysis, offering an opportunity for future research to reduce this burden and enhance scalability. These technologies, however, are still in their early stages of development. Their performance relies on the quality of captured food images, including image resolution, lighting conditions, and food presentation, as these factors can impact automated food identification and portion size estimation [18,24]. Establishing image quality standards may facilitate the integration of automated technologies with diet quality assessment, potentially reducing participant and researcher burden while maintaining acceptable accuracy [95].

## 5. Conclusions

As mobile applications are increasingly used for dietary monitoring by individuals and in clinical practice by healthcare providers, such as dietitians, validated methods for evaluating diet quality from images are needed. In this sample of Australian adults living with higher weight, the DQS demonstrated confirmed construct validity, with performance comparable to HEIFA-2013. Expected associations were observed with age, self-reported attention to health aspects of diet, and physical activity. Key factors associated with lower diet quality included lower attention to diet and higher total cholesterol, supporting the ability of the DQS to identify individuals most likely to benefit from dietary change.

These findings suggest that the DQS can be used to successfully evaluate diet quality directly from food images without requiring additional detailed information, such as nutrient intakes. This food-based approach to diet quality evaluation may facilitate the delivery of more practical and actionable feedback for personalised nutrition interventions by identifying specific food group patterns that individuals can target to improve their eating habits. Furthermore, its compatibility with image-based dietary records supports integration with automated dietary assessment tools and facilitates its use in large-scale public health monitoring. As this study was conducted in adults with higher weight, future research should evaluate the DQS in broader populations to assess its feasibility and acceptability in clinical practice settings. Additionally, future work should explore integration with AI-driven automated image analysis and apply the tool within longitudinal studies to track changes in diet quality over time.

## Figures and Tables

**Table 1:** Scoring framework for the DQS derived from the Australian Dietary Guidelines-2013, based on average daily intake.

Item Description	Minimum Score	Composite Scores	Maximum Score
**Adequacy components**			
Fruit	0 serves to <0.5 serves (0 points)	▪ ≥0.5 to <1.0 serves (2.5 points) ▪ ≥1.0 to <1.5 serves (5 points) ▪ ≥1.5 to <2.0 serves (7.5 points)	≥2 serves (10 points)
Vegetables	0 serves to <1.0 serves (0 points)	▪ ≥1.0 to <2.0 (2 point) ▪ ≥2.0 to <3.0 serves (4 points) ▪ ≥3.0 to <4.0 serves (6 points) ▪ ≥4.0 to <5.0 serves (8 points)	≥5 serves (10 points)
Variety of fruits	0 types (0 points)	▪ 1 type (1 point) ▪ 2 types (2 points) ▪ 3 types (3 points) ▪ 4 types (4 points)	≥5 types (5 points)
Variety of vegetables	0 types (0 points)	▪ 1 type (1 point) ▪ 2 types (2 points) ▪ 3 types (3 points) ▪ 4 types (4 points)	≥5 types (5 points)
Milk, yoghurt and cheese	<0.5 serves (0 points)	▪ ≥0.5 to <1.0 serves (2 points) ▪ ≥1 to <1.5 serves (4 points) ▪ ≥1.5 to <2.0 serves (6 points) ▪ ≥2 to <2.5 serves (8 points)	≥2.5 serves (10 points)
Plain water	<1 cup (0 points)	▪ ≥1 to 3 cups (2 points) ▪ ≥3 to 5 cups (6 points) ▪ ≥5 to <8 cups (women; 8 points) ▪ ≥5 to <10 cups (men; 8 points)	▪ ≥8 cups (women; 10 points) ▪ ≥10 cups (men; 10 points)
**Optimal range components**			
Lean meat, poultry, fish, eggs, nuts, seeds, legumes	Women: <0.5 serves or >2.5 serves (0 points)	Women: ▪ ≥0.5 to <1.0 (2 points) ▪ ≥1.0 to <1.5 (4 points) ▪ ≥1.5 to <2.0 (6 points) ▪ ≥2.0 to <2.5 (8 points)	Women: 2.5 serves (10 points)
	Men: <1.0 serves or >3 serves (0 points)	Men: ▪ ≥1.0 to <1.5 serves (2 points) ▪ ≥1.5 to <2.0 serves (4 points) ▪ ≥2.0 to <2.5 serves (6 points) ▪ ≥2.5 to <3.0 serves (8 points)	Men: 3 serves (10 points)
Grain (cereal) foods	<1.0 serves or >6.0 serves (0 points)	▪ ≥1.0 to <2.0 serves (1 point) ▪ ≥2.0 to <3 serves (2 points) ▪ ≥3.0 to <4 serves (5 points) ▪ ≥4.0 to <5 serves (6 points) ▪ ≥5.0 to <6 serves (8 points)	6.0 serves (10 points)
**Moderation components**			
Discretionary foods	≥2.75 serves (0 points)	▪ ≥1.75 to <2.75 serves (2 points) ▪ ≥0.75 to <1.75 serves (4 points) ▪ >0 to <0.75 serves (8 points)	0 serves (10 points)
Sugar-sweetened beverages	≥2 serves (0 points)	▪ ≥1.25 to <2 serves (2 points) ▪ ≥0.5 to <1.25 serves (4 points) ▪ >0 to <0.50 serves (8 points)	0 serves (10 points)
Alcoholic beverages	≥2 serves (0 points)	▪ ≥1.25 to <2 serves (2 points) ▪ ≥0.5 to <1.25 serves (4 points) ▪ >0 to <0.50 serves (8 points)	0 serves (10 points)

**Table 2:** Comparison of the scoring framework between the HEIFA-2013 and DQS.

Scoring Framework Features	HEIFA-2013 ^1^	DQS
**Scoring structure**		
Guidelines	▪ Australian Dietary Guidelines-2013	▪ Australian Dietary Guidelines-2013
Food or Nutrient-based	▪ Food- and nutrient-based	▪ Food-based
Total number of scored components	▪ 15	▪ 11
Food groups	▪ 6 (fruit, vegetable, cereal/bread, milk and alternatives, meat and protein/alternatives, discretionary foods)	▪ 6 (fruit, vegetable, grain (cereal), milk and alternatives, meat and protein/alternatives, discretionary foods)
Dietary variety	▪ 2 (fruit, vegetable)	▪ 2 (fruit, vegetable)
Grain quality	▪ 1 (wholegrain proportion)	
Beverages	▪ 2 (water, alcohol)	▪ 3 (water, alcohol, sugar-sweetened beverages)
Nutrients	▪ 4 (saturated fat, unsaturated fat, added sugars, sodium, alcohol)	
Scoring	▪ Components scored 0–5, or 0–10 ▪ 0–100 total score	▪ Components scored 0–5, or 0–10 ▪ 0–100 total score
**Scoring approach**		
	▪ Foods disaggregated into ingredients using fixed composition ratios, with ingredients assigned to food groups. Ingredient weights converted to food group serves using Australian Dietary Guidelines 2013 standards	▪ Food group servings determined directly from the Australian Health Survey pre-calculated values per 100 g of foods consumed
**Scoring processing**		
Proportion-based (e.g., %)	▪ Wholegrain (proportion of total wholemeal/grain bread consumed relative to total bread) ▪ Added sugars (% of energy) ▪ Saturated fat (% of energy) ▪ Water (% of total beverages)	
Intake quantity-based (e.g., serves/day)	▪ All remaining components	▪ All components
**Scoring direction**		
Adequacy components	▪ Fruit (quantity & variety) ▪ Vegetable (quantity & variety) ▪ Water ▪ Cereal/bread ▪ Wholegrains ▪ Milk and alternatives ▪ Meat and protein alternatives ▪ Unsaturated fat	▪ Fruit (quantity & variety) ▪ Vegetable (quantity & variety) ▪ Water
Optimal range components		▪ Grain (cereal) foods ▪ Meat and protein alternatives
Moderation components	▪ Discretionary foods ▪ Alcohol ▪ Sodium ▪ Added sugars ▪ Saturated fat	▪ Discretionary foods ▪ SSB ▪ Alcohol

^1^ Information regarding the scoring framework for HEIFA-2103 was retrieved from Roy et al. [10]. Refinements were made to water intake calculations, alcohol criteria, alignment with AUSNUT2011-13 food codes and the discretionary flag and precision of the scoring scales [58,59].

**Table 3:** Participant characteristics (*n* = 260).

Variable	Description	Men (*n* = 75)	Women (*n* = 185)	Total (*n* = 260)
		Mean ± SD	Mean ± SD	Mean ± SD
Age	Years	47 ± 12	48 ± 12	47.8 ± 11.7
Anthropometric measures				
Weight	kg	108.9 ± 15.9	95.5 ± 13.7	99.4 ± 15.6
BMI	kg/m^2^	34.5 ± 4.5	35.15 ± 4	35.0 ± 4.2
Body fat	%	31.7 ± 5.2	43.2 ± 3.5	39.9 ± 6.6
Clinical health markers				
Total cholesterol	mmol/L	5.2 ± 1.1 *	5.4 ± 1.0 *	5.3 ± 1.0 *
HDL-Cholesterol	mmol/L	1.2 ± 0.3	1.5 ± 0.3	1.4 ± 0.3
LDL-Cholesterol	mmol/L	3.2 ± 0.9 *	3.3 ± 0.9 *	3.3 ± 0.9 *
Non-HDL Cholesterol	mmol/L	4.0 ± 1.1 *	3.9 ± 1.0 *	4.0 ± 1.0 *
Cholesterol-HDL Ratio	ratio	4.6 ± 1.2 *	3.9 ± 1.0 *	4.1 ± 1.1 *
Triglycerides	mmol/L	1.8 ± 1.0	1.4 ± 0.8	1.5 ± 0.9
HbA1C	%	5.7 ± 0.7 *	5.6 ± 0.5 *	5.6 ± 0.6 *
Fasting glucose	mmol/L	5.5 ± 0.9 *	5.3 ± 0.9 *	5.3 ± 0.9 *
Sociodemographic		*n* (%)	*n* (%)	*n* (%)
Ethnicity	White	55 (28.2%)	140 (71.8%)	195 (75.6%)
	Other ^1^	20 (31.7%)	43 (68.3%)	63 (24.4%)
IRSAD ^2^	1–2	3 (50.0%)	3 (50.0%)	6 (2.3%)
	3–4	16 (28.1%)	41 (71.9%)	57 (21.9%)
	5–6	22 (29.3%)	53 (70.7%)	75 (28.8%)
	7–8	8 (32.0%)	17 (68.0%)	25 (9.6%)
	9–10	26 (26.8%)	71 (73.2%)	97 (37.3%)
Education	Senior Secondary Certificate of Education	5 (17.9%)	23 (82.1%)	28 (10.9%)
	Apprenticeship/Diploma	20 (31.7%)	43 (68.3%)	63 (24.4%)
	University bachelor’s degree or higher	49 (29.3%)	118 (70.7%)	167 (64.7%)
Household income	<$29,999–$99,999	15 (21.4%)	55 (78.6%)	70 (29.7%)
	$100,000–$199,999	36 (32.1%)	77 (68.1%)	113 (47.9%)
	≥$200,000	17 (25.0%)	36 (21.4%)	53 (22.5%)
IPAQ Category ^3^	Low/Inactive	38 (30.4%)	87 (69.6%)	125 (48.1%)
	Moderate	20 (23.5%)	65 (76.5%)	85 (32.7%)
	High/Active	17 (34.0%)	33 (66.0%)	50 (19.2%)
Smoker status ^4^	Current smoker	4 (40.0%)	6 (60.0%)	10 (3.9%)
	Past smoker	36 (34.6%)	68 (65.4%)	104 (40.2%)
	Never smoked	35 (24.1%)	110 (75.9%)	145 (56.0%)
Food sufficiency	Enough of the kinds of food wanted	61 (29.3%)	147 (70.7%)	208 (82.5%)
	Enough, but not always the kinds wanted	11 (25.0%)	33 (75.0%)	44 (17.5%)
Cooking skills	Can boil an egg, or BBQ meat or heat frozen meals	6 (60.0%)	4 (40.0%)	10 (3.9%)
	Can cook basic meat and 3 veg type meals	21 (48.8%)	22 (51.2%)	43 (16.9%)
	Can cook a wide variety of meals	29 (24.6%)	89 (75.4%)	118 (46.5%)
	Can cook almost anything	19 (22.9%)	64 (77.1%)	83 (32.7%)
Attention paid to health aspects of diet	Pay a lot of attention to the health aspects of food	2 (10.5%)	17 (89.5%)	19 (7.7%)
	Take a bit of notice of the health aspects of food	50 (27.8%)	130 (72.2%)	180 (72.6%)
	Don’t think much or don’t think at all	19 (38.8%)	30 (61.2%)	49 (19.8%)
Past assisted weight loss attempts	Never	37 (49.3%)	38 (50.7%)	75 (29.0%)
	1–2	25 (25.0%)	75 (75.0%)	100 (38.6%)
	3–5	11 (20.8%)	42 (79.2%)	53 (20.5%)
	6 and more	2 (6.5%)	29 (93.5%)	31 (12.0%)
Past unassisted weight loss attempts	Never	9 (37.5%)	15 (62.5%)	24 (9.3%)
	1–2	20 (29.4%)	48 (70.6%)	68 (26.3%)
	3–5	25 (35.7%)	45 (64.3%)	70 (27.0%)
	6–10	11 (23.4%)	36 (76.6%)	47 (18.1%)

^1^ Other ethnicity refers to Asian, Aboriginal and/or Torres Strait Islander, Pacific Islander, Black and/or African American, and Mixed/Multiple Ethnicities; ^2^ IRSAD, Index of Relative Socio-Economic Advantage and Disadvantage; ^3^ IPAQ, International Physical Activity Questionnaire; ^4^ smoker status is inclusive of vaping. * Indicates mean value above the clinical reference range.

**Table 4:** Mean diet quality scores for HEIFA-2013 and DQS, inclusive of dietary component scores and total scores.

Dietary Component	HEIFA-2013		DQS	
	Mean Score	Maximum Points	Mean Score	Maximum Points
**Fruit and vegetables**				
Fruit	2.3 ± 2.5	5 points	2.5 ± 2.9	10 points
Fruit Variety	1.2 ± 1.5	5 points	0.6 ± 0.9	5 points
Vegetables and legumes/beans	3.0 ± 1.7	5 points	4.8 ± 2.8	10 points
Vegetable Variety	1.0 ± 0.7	5 points	3.0 ± 1.4	5 points
**Grain (cereal) foods**				
Cereal/bread	2.7 ± 1.1	5 points	-	-
Wholegrains	1.2 ± 1.3	5 points	-	-
Grain (cereal)	-	-	4.1 ± 2.7	10 points
**Other foods**				
Milk, yoghurt, cheese and/or alternatives	4.8 ± 2.6	10 points	4.7 ± 3.2	10 points
Meats, poultry, fish, eggs, tofu, nuts and seeds and legumes/beans	6.4 ± 2.3	10 points	3.5 ± 3.1	10 points
Discretionary Foods	5.6 ± 2.8	10 points	1.2 ± 2.0	10 points
**Beverages**				
Alcohol	4.6 ± 0.9	5 points	8.7 ± 2.4	10 points
Sugar-sweetened beverages	-	-	8.3 ± 2.5	10 points
Plain water	1.9 ± 2.0	5 points	6.0 ± 3.2	10 points
**Nutrients**				
Sugar	9.5 ± 1.3	10 points	-	-
Sodium	4.4 ± 2.9	10 points	-	-
Unsaturated fat	4.6 ± 0.7	5 points	-	-
Saturated fat	1.3 ± 1.3	5 points	-	-
**Overall Diet Quality Score**	52.0 ± 8.6	100 points	47.4 ± 8.7	100 points

**Table 5:** Comparison of HEIFA-2013 and DQSs across categorical sociodemographic variables.

Variable	Description	*n* = 260	HEIFA-2013	DQS
			Mean ± SD	Mean ± SD
Gender	Male	75	50.3 ± 7.0	49.0 ± 8.4
	Female	185	52.7 ± 9.0	46.7 ± 8.7
Ethnicity	White	195	52.1 ± 8.8	47.9 ± 9.1
	Other ^1^	63	51.7 ± 8.0	45.9 ± 7.1
IRSAD ^2^	1–2	6	52.9 ± 8.5	45.5 ± 8.3
	3–4	57	55.1 ± 7.7	55.9 ± 11.3
	5–6	75	52.7 ± 8.6	47.7 ± 7.6
	7–8	25	50.2 ± 8.3	46.0 ± 8.2
	9–10	97	51.6 ± 7.8	49.7 ± 8.2
Education	Senior secondary certificate of education	28	53.0 ± 8.9	47.0 ± 9.2
	Apprenticeship/diploma	63	51.5 ± 9.3	46.8 ± 8.3
	University bachelor’s degree or higher	167	52.5 ± 8.4	47.7 ± 8.8
Household income	<$29,999–$99,999	70	50.9 ± 8.7	46.6 ± 8.9
	$100,000–$199,999	113	53.3 ± 8.1	48.2 ± 9.0
	≥$200,000	53	52.1 ± 8.9	46.9 ± 8.2
IPAQ category ^3^	Low/Inactive	125	51.0 ± 8.6	46.2 ± 8.7
	Moderate	85	53.8 ± 8.9	49.7 ± 8.9
	High/Active	50	51.6 ± 7.6	46.2 ± 7.3
Smoker status ^4^	Current smoker	10	50.5 ± 7.9	44.9 ± 6.7
	Past smoker	104	52.4 ± 8.2	48.4 ± 8.8
	Never smoked	145	51.8 ± 8.9	46.8 ± 8.6
Food sufficiency	Enough of the kinds of food wanted	208	52.7 ± 8.6	48.0 ± 8.6
	Enough, but not always the kinds wanted	44	50.1 ± 8.2	44.9 ± 9.1
Cooking skills	Can boil an egg, or BBQ meat or heat frozen meals	10	49.4 ± 9.2	44.8 ± 9.5
	Can cook basic meat and 3 veg type meals	43	50.1 ± 8.7	46.2 ± 8.9
	Can cook a wide variety of meals	118	52.8 ± 8.6	47.6 ± 9.1
	Can cook almost anything	83	52.4 ± 8.2	48.0 ± 7.7
Attention paid to health aspects of diet	Pay a lot of attention to the health aspects of food	19	51.3 ± 7.5	55.4 ± 8.6
	Take a bit of notice of the health aspects of food	180	52.7 ± 8.5	47.6 ± 8.8
	Don’t think much or don’t think at all	49	49.2 ± 8.3	45.1 ± 8.2
Past assisted weight loss attempts	Never	75	51.1 ± 8.1	47.9 ± 8.8
	1–2	100	51.9 ± 8.9	47.1 ± 8.6
	3–5	53	53.9 ± 8.0	46.8 ± 8.0
	6 and more	31	51.3 ± 9.6	47.9 ± 9.9
Past unassisted weight loss attempts	Never	24	49.2 ± 7.5	46.8 ± 9.2
	1–2	68	52.2 ± 8.5	47.6 ± 9.1
	3–5	70	52.3 ± 7.9	47.7 ± 8.4
	6–10	47	50.6 ± 9.4	47.6 ± 7.8

^1^ Other ethnicity refers to Asian, Aboriginal and/or Torres Strait Islander, Pacific Islander, Black and/or African American, and Mixed/Multiple Ethnicities; ^2^ IRSAD, Index of Relative Socio-Economic Advantage and Disadvantage; ^3^ IPAQ, International Physical Activity Questionnaire; ^4^ smoker status is inclusive of vaping.

**Table 6:** Variables associated with being classified in the lowest diet quality tertile by HEIFA-2013 and DQS.

Variables	Odds-Ratio	95% Confidence Interval	*p*-Value
Total cholesterol level (mmol/L)	1.385	1.031–1.861	0.031
Age	0.957	0.931–0.984	0.002
IPAQ Category			0.047
Low/Inactive (Reference)	1		
Moderate	0.435	0.225–0.843	0.014
High/Active	0.748	0.338–1.658	0.475
Attention paid to the health aspects of diet			0.030
Pay a lot of attention (Reference)	1		
Bit of notice	12.480	1.571–99.157	0.017
Don’t think too much	18.117	2095–156.653	0.008

## Data Availability

The data presented in this study are available on reasonable request from the corresponding author.

## Abbreviations

The following abbreviations are used in this manuscript:

AI	artificial intelligence
AUSNUT	Australian Food and Nutrient Database
BMI	body mass index
BMR	basal metabolic rate
chat2	Connecting Health and Technology 2
CI	confidence interval
DQI	diet quality index
DQS	Diet Quality Score
EI	energy intake
HSDI	Healthy and Sustainable Diet Index
HEIFA-2013	Healthy Eating Index for Australians 2013
IPAQ	International Physical Activity Questionnaire
IRSAD	Index of Relative Socio-Economic Advantage and Disadvantage
MET	Metabolic Equivalent of Task
mFR™	mobile Food Record
NNPAS	National Nutrition and Physical Activity Survey
OR	odds ratio
SSBs	sugar sweetened beverages
